# Predictors predisposing to orocutaneous fistula occurrence following free flap reconstruction

**DOI:** 10.3389/fonc.2022.947643

**Published:** 2022-07-18

**Authors:** Wenlu Li, Shuang Wu, Junhui Yuan, Fan Meng, Chunmiao Xu, Hailiang Li

**Affiliations:** ^1^ Department of Stomatology, The Affiliated First Hospital of Zhengzhou University, Zhengzhou University, Zhengzhou, China; ^2^ Department of Radiology, The Affiliated Cancer Hospital of Zhengzhou University & Henan Cancer Hospital, Zhengzhou, China

**Keywords:** free flap, tongue, floor of the mouth, orocutaneous fistula, cachexia, system design

## Abstract

**Objectives:**

To explore the possible risk factors of orocutaneous fistula (OCF) development in free flap reconstruction of the tongue/floor of the mouth (TFOM).

**Methods:**

Data of patients who underwent free flap reconstruction of the TFOM were retrospectively analyzed. The association between clinicopathologic variables and OCF occurrence was analyzed using univariate and multivariate analyses.

**Results:**

Altogether, 469 patients were enrolled. OCF occurred in 43 patients with a rate of 9.2%. The univariate analysis revealed the negative effects of smoking, preoperative albumin level, cachexia, T4 stage, neck dissection, entire resection of the floor of the mouth (FOM), segmental mandibulectomy, and surgical site infection on OCF occurrence. The multivariate analysis confirmed the independence of cachexia (p<0.001, 4.386[1.883–9.472]), tumor stage (p<0.001, 2.738[1.482–6.629]), entire FOM resection (p<0.001, 6.332[2.110–14.432]), and surgical site infection (p<0.001, 5.376[1.998–11.218]) in affecting the OCF development.

**Conclusions:**

OCF development following free flap reconstruction of the TFOM was relatively uncommon, but significantly associated with presence of cachexia, T4 stage, entire FOM resection, and surgical site infection.

## Introduction

Squamous cell carcinoma (SCC) in the tongue/floor of the mouth (TFOM), whose features include lymph node metastasis and local invasion, is the most common malignancy in the oral cavity. At the initial diagnosis, the majority of these tumors have an advanced stage (1, 2). Extensive soft or bone tissue resection is required, and free flap reconstruction is generally the preferred method (3); however, postoperative complications may develop frequently (4). One complication is orocutaneous fistula (OCF), which could cause significantly negative effects such as delays in adjuvant therapy initiation and oral feeding and voice rehabilitation, as well as increase in treatment costs, length of hospital stay, and risk of carotid blowout (5–7). Nowadays, methods for treating OCF include conventional wound care, negative pressure wound therapy, and surgical management (8–11).

A previous study has reported patient-related, tumor-related, and treatment-related factors in predicting fistula development after a larynx/hypopharynx surgery (12). However, considering the fact that there is larger and more dead space after a TFOM operation, there might be unique predictors for OCF occurrence in TFOM surgery.

However, studies regarding the predictors of OCF formation in oral surgery are rare (13–15). Dawson et al. (13) may be the first to analyze this issue and have reported an overall incidence of OCF occurrence of 11% in patients with different etiologies, such as cancer, ameloblastoma, osteoradionecrosis, and trauma. Moreover, previous chemoradiotherapy was the only significant factor increasing the possibility of OCF formation. Additionally, Girkar et al. (14) have reported a rate of 9% and identified that patients with surgical site infection (SSI) and bilateral neck dissection are at the maximum risk for developing OCF. No other similar literature is available for analyzing.

Therefore, this study aimed to explore the possible risk factors of OCF development in free flap reconstruction for TFOM SCC.

## Patients and methods

### Ethical considerations

The study was approved by The First Affiliated Hospital of Zhengzhou University institutional research committee, and all participants provided written informed consent. All methods and procedures were performed in accordance with the 1964 Helsinki Declaration.

### Patient selection

Medical records of adult patients with surgically treated oral SCC between January 2010 and January 2022 were retrospectively reviewed in a Tertiary Level Teaching Hospital. Patients with primary TFOM SCC and those with defects repaired with a free flap were included. Those with a history of other malignant diseases or prior chemoradiotherapy were excluded. Data on demographics; body mass index (BMI); systemic diseases (hypertension, diabetes mellitus, and cardiovascular and cerebrovascular diseases); thyroid stimulating hormone (TSH) levels; cachexia; tumor, nodes, metastases stage according to the 8^th^ American Joint Committee on Cancer classification; complications; and treatment were extracted and analyzed.

### Definitions of variables

The normal range of whole blood hemoglobin was 115–150 g/L for women and 130–145 g/L for men. The normal range of venous albumin was 35–55 g/L. The normal range of blood serum TSH was 0.35–5.5 IU/ml. Based on the official standards by the World Health Organization for Asians (16), obesity, overweight, normal weight, and underweight were defined as BMI ≥ 25 kg/m^2^, 23 kg/m^2^ ≤ BMI < 25 kg/m^2^, 18.5 kg/m^2^ ≤ BMI < 23 kg/m^2^, and BMI < 18.5 kg/m^2^, respectively. To simplify the analysis, the patients were classified into three groups with two BMI cutoffs of 23 kg/m^2^ and 18.5 kg/m^2^.

SSI was regarded as the occurrence of purulent drainage or an incision that spontaneously dehisces in a patient with fever and/or localized pain or tenderness or deep site infection detected by radiographic examination within 30 days after surgery (2).

Cachexia was regarded as the occurrence of weight loss > 2% in an individual already showing depletion or weight loss > 5% relative to the current body weight and height or skeletal muscle mass (17).

### Treatment

Resection with a margin of at least 1 cm with lower lip splitting was attempted in all tumors. Excision methods of primary focus included hemi, total, or subtotal glossectomy combined with resection of the floor of the mouth (FOM). Segmental mandibulectomy was performed if apparent mandible involvement was detected intraoperatively or preoperatively. Neck dissection was performed for all patients, level I to III/IV dissection was for a cN0 neck, and level I to V dissection for a cN+ neck. The type of free flap was considered mostly based on the defect size and range.

For the first 24 h postoperatively, the flap was monitored hourly and then every 4 h for the next 72 h. Cephalosporin was administered intravenously 30 min before incision, and continued for at least 3–5 days postoperatively. Before patients were able to feed orally, nasogastric nutrition was administered routinely (18).

### OCF

OCF was generally verified by a swallow study or dye test when there was combined clinical detection of a visible breach in the intraoral suture line, a foul odor emanating from the oral cavity, congestion and/or edema of the neck flap, collection in the neck, fever, turbid drain, and leukocytosis (13, 14).

In our cancer center (a Tertiary Level Teaching Hospital), the preferred method of treating an OCF was commonly conventional wound care consisting of wound flushing with saline and hydrogen peroxide as well as compressive dressings. If the fistula was enormous determined by experience or the closure time was excessively long, negative pressure wound therapy and secondary surgery were selectively performed.

### Statistical analysis

The relationship between OCF occurrence and clinicopathologic variables was firstly analyzed using the Chi-square test (univariate analysis). Then, the significant factors in the univariate analyses were included in the multivariate analysis (binomial logistic regression). All statistical analyses were performed using SPSS 20.0, and a p<0.05 was considered significant.

## Results

Overall, 469 patients, comprising 357 men and 112 women, were included for analysis. The median age was 55 (range: 36–76) years. Smoking and drinking were recorded in 278 and 124 patients, respectively. Hypertension, diabetes mellitus, and cardiovascular and cerebrovascular diseases occurred in 111, 56, and 77 patients, respectively. Low hemoglobin and albumin levels were present in 103 and 88 patients, respectively. Low, normal, and high TSH levels were recorded in 46, 372, and 51 patients, respectively. Cachexia was noted in 189 patients.

Tumor stages were distributed as T2 in 117 patients, T3 in 201 patients, and T4 in 151 patients. Neck stages were distributed as N0 in 276 patients and N+ in 193 patients. Hemi, subtotal, and total glossectomies were performed in 234, 156, and 79 patients, respectively. Partial and entire resections of the FOM were performed in 252 and 129 patients, respectively. Segmental mandibulectomy was performed in 168 patients. Unilateral and bilateral neck dissections were performed in 353 and 116 patients, respectively. Radial forearm free (RFF) flap, anterolateral thigh flap, and free fibula osteocutaneous (FFO) flap reconstructions were performed in 207, 154, and 108 patients, respectively.

SSI occurred in 85 patients. Vascular crisis developed in 45 flaps, and exploratory operation successfully salvaged 25 flaps. In the remaining 20 cases, a local flap was used for 10 patients, and a major pectoralis myocutaneous flap was used for ten patients (**Table 1**).

**Table 1:** Clinical and pathologic features in the 469 patients.

Variable	Number (%)
Age	
<55	234 (49.9%)
≥55	235 (50.1%)
Sex	
Male	357 (76.1%)
Female	112 (23.9%)
Current Smoker^	278 (59.3%)
Current Drinker^&^	124 (26.4%)
Preoperative whole blood hemoglobin level	
Low	103 (22.0%)
Normal	366 (78.0%)
Preoperative venous albumin level	
Low	88 (18.8%)
Normal	381 (81.2%)
Hypertension	111 (23.7%)
Diabetes mellitus	56 (11.9%)
Cardiovascular and cerebrovascular disease	77 (16.4%)
Preoperative serum TSH level	
Low	46 (9.8%)
Normal	372 (79.3%)
High	51 (10.9%)
Cachexia	189 (40.3%)
Tumor stage	
T2	117 (24.9%)
T3	201 (42.9%)
T4	151 (32.2%)
Neck dissection	
Unilateral	353 (75.3%)
Bilateral	116 (24.7%)
Flap type	
Radial free forearm flap	207 (44.1%)
Anterolateral thigh flap	154 (32.8%)
Free fibula osteocutaneous flap	108 (23.0%)
Resection of the FOM*	381 (81.2%)
Segmental mandibulectomy	168 (35.8%)
Flap failure	20 (4.2%)
Surgical site infection	85 (18.1%)
Orocutaneous fistula	43 (9.2%)

*FOM: the floor of the mouth;

^Current smoker was defined as one who had smoked at least 20 cigarettes per day for at least 5 years.

^&^Current drinker was defined as one who had drunk wine at least once per day for at least 5 years.

### OCF occurrence

OCF occurred in 43 patients with a rate of 9.2%. The mean size of OCF was 2.8 ± 1.3 cm. The incidence of OCF development in patients with low and normal preoperative albumin levels was 15.9% and 7.6%, respectively, with a significant difference (p=0.015). Smokers had a rate of OCF formation of 11.9%, it was significantly higher than 5.2% in non-smokers (p=0.014). Patients with cachexia had a significantly higher OCF formation rate than those without cachexia (12.2% vs 5.3%, p=0.003). Patients with T4 tumors had a significantly higher OCF incidence than patients with T2/3 tumors (16.6% vs 5.7%, p<0.001). The bilateral neck dissection group had a significantly higher OCF development rate than the unilateral neck dissection group (13.8% vs 7.6%, p=0.047). Patients who underwent an entire FOM resection had a significantly higher OCF occurrence rate than patients who underwent no or partial FOM resection (23.2% vs 3.8%, p<0.001). Segmental mandibulectomy indicated an OCF occurrence rate of 13.1%, which was significantly higher than 7.0% in non-segmental mandibulectomy group (p=0.028). A significant difference was observed in the rate of OCF development in the SSI group and the non-SSI group at 20.0% and 6.8%, respectively (p<0.001). No other significant factors were associated with OCF development (**Table 2**).

**Table 2:** Univariate analysis of the association between clinicopathologic variables and orocutaneous fistula (OCF) development.

Variable	OCF		p
	Yes (n=43)	No (n=426)	
Age			
<55	19	215	
≥55	24	211	0.432
Sex			
Male	35	322	
Female	8	104	0.395
Current Smoker^			
No	10	181	
Yes	33	245	0.014
Current Drinker^&^			
No	32	313	
Yes	11	113	0.894
Preoperative whole blood hemoglobin level			
Low	13	90	
Normal	30	336	0.169
Preoperative venous albumin level			
Low	14	74	
Normal	29	352	0.015
Hypertension			
No	34	324	
Yes	9	102	0.658
Diabetes mellitus			
No	39	374	
Yes	4	52	0.576
Cardiovascular and cerebrovascular disease			
No	38	354	
Yes	5	72	0.374
Cachexia			
No	20	360	
Yes	23	166	0.003
Preoperative serum TSH level			
Low	3	43	
Normal	35	337	
High	5	46	0.804
Tumor stage			
T2+T3	18	300	
T4	25	126	<0.001
Neck dissection			
Unilateral	27	326	
Bilateral	16	100	0.047
Flap type			
Radial free forearm flap	19	188	
Anterolateral thigh flap	14	140	
Free fibula osteocutaneous flap	10	98	1.000
Resection of the tongue			
Hemi-glossectomy	20	214	
Subtotal glossectomy	15	141	
Total glossectomy	8	71	0.890
Resection of the FOM*			
No or partial	13	327	
Entire	30	99	<0.001
Segmental mandibulectomy			
No	21	280	
Yes	22	146	0.028
Flap failure			
No	41	408	
Yes	2	18	1.000
Surgical site infection			
No	26	358	
Yes	17	68	<0.001

*FOM: the floor of the mouth.

^Current smoker was defined as one who had smoked at least 20 cigarettes per day for at least 5 years.

^&^Current drinker was defined as one who had drunk wine at least once per day for at least 5 years.

In the subsequent multivariate analyses, cachexia (p<0.001, 4.386[1.883–9.472]), tumor stage (p<0.001, 2.738[1.482–6.629]), entire FOM resection (p<0.001, 6.332[2.110–14.432]), and SSI (p<0.001, 5.376[1.998–11.218]) were independently associated with increased risk of the occurrence of OCF (**Table 3**).

**Table 3:** Multivariate analysis of the association between clinicopathologic variables and orocutaneous fistula (OCF) development.

Variable	p	Odds ratio [95%CI]
Current Smoker^	0.327	2.168[0.846-5.338]
Preoperative venous albumin level	0.174	3.217[0.725-8.114]
Cachexia	<0.001	4.386[1.883-9.472]
Tumor stage	<0.001	2.738[1.482-6.629]
Neck dissection	0.683	3.827[0.884-8.276]
Segmental mandibulectomy	0.432	2.117[0.632-5.174]
Entire resection of the FOM*	<0.001	6.332[2.110-14.432]
Surgical site infection	<0.001	5.376[1.998-11.218]

*FOM: the floor of the mouth.

^Current smoker was defined as one who had smoked at least 20 cigarettes per day for at least 5 years.

### OCF treatment

All OCFs were initially treated by wound washing with normal saline and hydrogen peroxide solution, and a gauze with iodine dressing was placed within the fistula to stimulate the development of fresh granulation tissue. Oral feeding was prohibited but was replaced by nasal feeding nutrition until total recovery of the fistula. Thirty OCFs achieved total recovery by the single use of conventional wound care, and the median duration for total recovery was 20 days with a range from 4 days to 47 days. The remaining thirteen OCFs were treated successfully by local flaps including seven sliding advancement flaps and six rotation flaps, and no secondary OCF formation was observed.

## Discussion

The most important finding in this study was that OCF development following free flap reconstruction for TFOM was relatively uncommon, but was significantly associated with the presence of cachexia, T4 stage, entire FOM resection, and SSI. Although conventional wound care alone could treat most OCFs, it was time-consuming.

Cachexia is associated with inadequate food intake, metabolic derangement, inactivity, loss of muscle mass, and weight loss in patients with advanced malignant tumors (17). More toxicities, treatment gaps, and postoperative complications as well as poorer disease-free survival are generally contributed by cachexia (19). Fukuta et al. (20) prospectively enrolled 98 patients with gastric or colorectal cancer, and 22.4% of those were diagnosed with cachexia; they discovered that the cachexia group had a significantly longer postoperative length of stay than the non-cachexia group. Similarly, Mason et al. (21) analyzed the short-term effect of cachexia on postoperative complications, and have reported that adverse events tended to occur in 64.3% of patients with cachexia, a rate that was significantly higher than that in those without cachexia. A study focusing on clinical impact of cachexia in patients with head and neck cancer undergoing chemoradiotherapy by Hayashi et al. (22) described that cachexia could play a negative role in all adverse events. Regarding grade 3/4 adverse events, a significant difference in the incidence of anemia, neutropenia, and leukopenia was observed between patients with and without cachexia. To the best of our knowledge, this study may be the first to report the importance of cachexia in causing OCF development, which could be explained by the impairing would healing capacity in patients with cachexia, it might be reflected by that cachexia patients were at higher risk of low preoperative venous blood albumin levels. This finding provides a reliable theoretical basis for decreasing OCF occurrence by improving patients’ nutritional status.

Tumor stage and entire FOM resection were both predictors and are also significantly associated with OCF occurrence. A T4 TFOM tumor generally indicated entire FOM resection with the help of lower lip splitting for complete disease clearance (23), then a large dead space could be expected; the importance of its obliteration had been emphasized by Al Deek et al. (6). The authors commented that the dead space between the hyoid bone and the mandible should be reconstructed with viable tissue to prevent OCF occurrence, which was occupied by suprahyoid muscles originally. Importantly, after establishing perfusion, reliability of the part of the flap inserted in the dead space should be re-checked, especially when there was an excess muscle resected or an orientation change during the flap inset. The viewpoint would also be supported by our results. Similarly, in a report by Girkar et al. (14), the rate of OCF development in patients with TFOM SCC and other subsites SCC was 15% and 7% independent of the used flap type, respectively, and the difference was significant. In our opinion, during the recovery period, motion of the reconstructed TFOM still occurred owing to the stimulation of saliva despite prohibition of oral intake. Therefore, in cases of an imperfect flap design, these movements could impair the water-tight closure directly, which caused accumulation of saliva at the TFOM. Thus, to minimize the possibility of suture dehiscence and tearing, tension must be distributed by multilayer mattress sutures that should be used to reduce tension.

Negative effects of SSI were also apparent, although they were common following free flap reconstruction and could develop in approximately 30% of patients (24). Both patient-related and treatment-related factors were related to SSI, including poor oral hygiene, poor nutritional status, prolonged operative time, type of reconstruction, faulty surgical techniques, and resection range. Šifrer et al. (25) recently described in their study of 48 patients with SSI that 38 patients suffered from pharyngocutaneous fistula, although in the patients without SSI, the rate of pharyngocutaneous fistula was only 18.2%. Moreover, the authors emphasize the importance of SSI timing, which should be started on the 5th postoperative day in the fistula group and on the 7th postoperative day in the control group. Andrades et al. (26) have reported that fistulas occurred in 28.8% of 104 patients undergoing RFF flap for hypopharyngeal reconstruction, although the incidence significantly increased in patients with SSI. Our results were also consistent with the finding. Generally, fistula and infections coexist, resulting in a vicious cycle where one may lead to the other (15). A fistula almost always presents as an infection initially, timely anti-infection treatment is essential for decreasing the OCF development. In the other hand, early detection of an OCF could be achieved by a swallow study or dye test from infected sites.

Negative effects of previous chemoradiotherapy and bilateral neck dissection have been reported in other literature (13, 14). On one hand, indication for bilateral neck dissection midline across the primary tumor or pathologically proven lymph nodes in the contralateral neck is related to an advanced stage disease, requiring extensive soft or bone tissue resection; increased surgery duration and intraoperative bleeding are expected. On the other hand, tissue fibrosis easily results from previous chemoradiotherapy and conversely causes poor wound healing (27). These aspects might help to predict the likelihood of OCF development in patients with the two features. However, this study failed to identify a positive association, and this might be due to the different study design and enrollment criteria. It could be noted that current OCF formulation rate is a little higher than that (7.7%) reported by Tassone et al. (27) who also enrolled patients with previous irradiation, there are at least three aspects contributing to the difference, first, all patients in current study received a lip split, second, the two studies have different inclusion criteria, third, previously radiated patients may not have had extensive neck surgery at the time of their salvage resections.

Segmental mandibulectomy is a reliable method for treating cases involving the mandible. Free flap reconstruction is commonly performed to maintain better appearance and functional results. However, short-term and long-term complications of this procedure, such as SSI and pathologic fracture/exposure of the mandible, have been previously reported (28, 29). However, in this study, we failed to identify the effects of segmental mandibulectomy on OCF occurrence. At least two aspects can explain this: firstly, the dead space between the mandible and hyoid disappeared after segmental mandibulectomy; secondly, no flap necrosis was observed in patients undergoing a FFO flap.

Smoking and drinking were the main risk factors for head and neck SCC, current study did not find the relationship between smoking and OCF development, but we still should emphasize the harmful effect of tobacco on postoperative complication (30). It was interesting to note that alcohol intake status did not appear to have a significant influence on OCF development in this study. Two aspects must be considered, on the one hand, even though East Asian patients consumed alcohol, but they were at a much lower risk for heavy alcoholism than people with functional aldehyde dehydrogenase (ALDH) genes which encodes one of the two enzymes primarily involved in alcohol metabolism (31). On the other hand, because of the high prevalence of ALDH2*2 allele among East Asian populations, East Asians may be more susceptible to the carcinogenic effect of alcohol, with most evidence coming from studies of esophageal cancer and head and neck cancer (32), however, perhaps, a more comprehensive study assessing the influence of alcohol intake on the postoperative TFOM reconstructive surgery complications in both Asian and non-Asian populations would be needed in the future.

Treating OCF requires longer time, which may take 1 month or longer (5). Conventional wound care, negative pressure wound therapy, and surgical treatment are the major treatment methods for OCF (6–11). Tian et al. (8), Inatomi et al. (10), and Khoo et al. (5) have verified the reliability of negative pressure wound therapy with a high success rate and shorter treatment time. This study might support the feasibility of conventional wound care with a high response rate, which was consistent with the finding of Andrades et al. (26).

Limitations in current study must be acknowledged, first, there was inherent bias within the retrospective study; second, both smoking and drinking status was not stratified based on the frequency and severity, it might cover up some interesting findings.

In summary, OCF development following free flap reconstruction for TFOM was relatively uncommon, although it was significantly associated with cachexia, T4 stage, entire FOM resection, and SSI. Although conventional wound care alone could cure most OCFs, it was time-consuming.

## Data availability statement

The original contributions presented in the study are included in the article/**Supplementary Material**. Further inquiries can be directed to the corresponding author.

## Ethics statement

The studies involving human participants were reviewed and approved by The First Affiliated Hospital of Zhengzhou University institutional research committee. The patients/participants provided their written informed consent to participate in this study.

## Author contributions

All the authors made the contribution in study design, manuscript writing, studies selecting, data analysis, study quality evaluating, and manuscript revising. All authors have read and approved the final manuscript.

## Conflict of interest

The authors declare that the research was conducted in the absence of any commercial or financial relationships that could be construed as a potential conflict of interest.

## Publisher’s note

All claims expressed in this article are solely those of the authors and do not necessarily represent those of their affiliated organizations, or those of the publisher, the editors and the reviewers. Any product that may be evaluated in this article, or claim that may be made by its manufacturer, is not guaranteed or endorsed by the publisher.
