# Supplementary figures and images for: Predictors predisposing to orocutaneous fistula occurrence following free flap reconstruction
Source: Front Oncol. 2022 Jul 18;12:947643. doi: 10.3389/fonc.2022.947643 (PMC9341452; doi:10.3389/fonc.2022.947643)

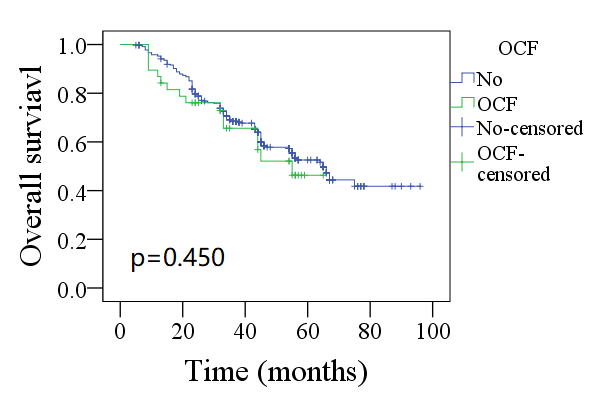

Supplement: Supplementary file 1 [file Image_1.tif]
